# Pre-Operative Trans-Arterial Embolization of a Hypervascular Bone Metastasis

**DOI:** 10.5334/jbsr.1694

**Published:** 2019-01-17

**Authors:** Benjamin Leenknegt, Filippo Pesapane, Dean Huang

**Affiliations:** 1 Ghent University Hospital, BE; 2 Università degli Studi di Milano, IT; 3 King’s College Hospital, GB

**Keywords:** Pathologic fracture, Bone metastasis, Renal Cell Carcinoma, Interventional Radiology, Trans-arterial embolisation

## Case Presentation

**Figure 1:**
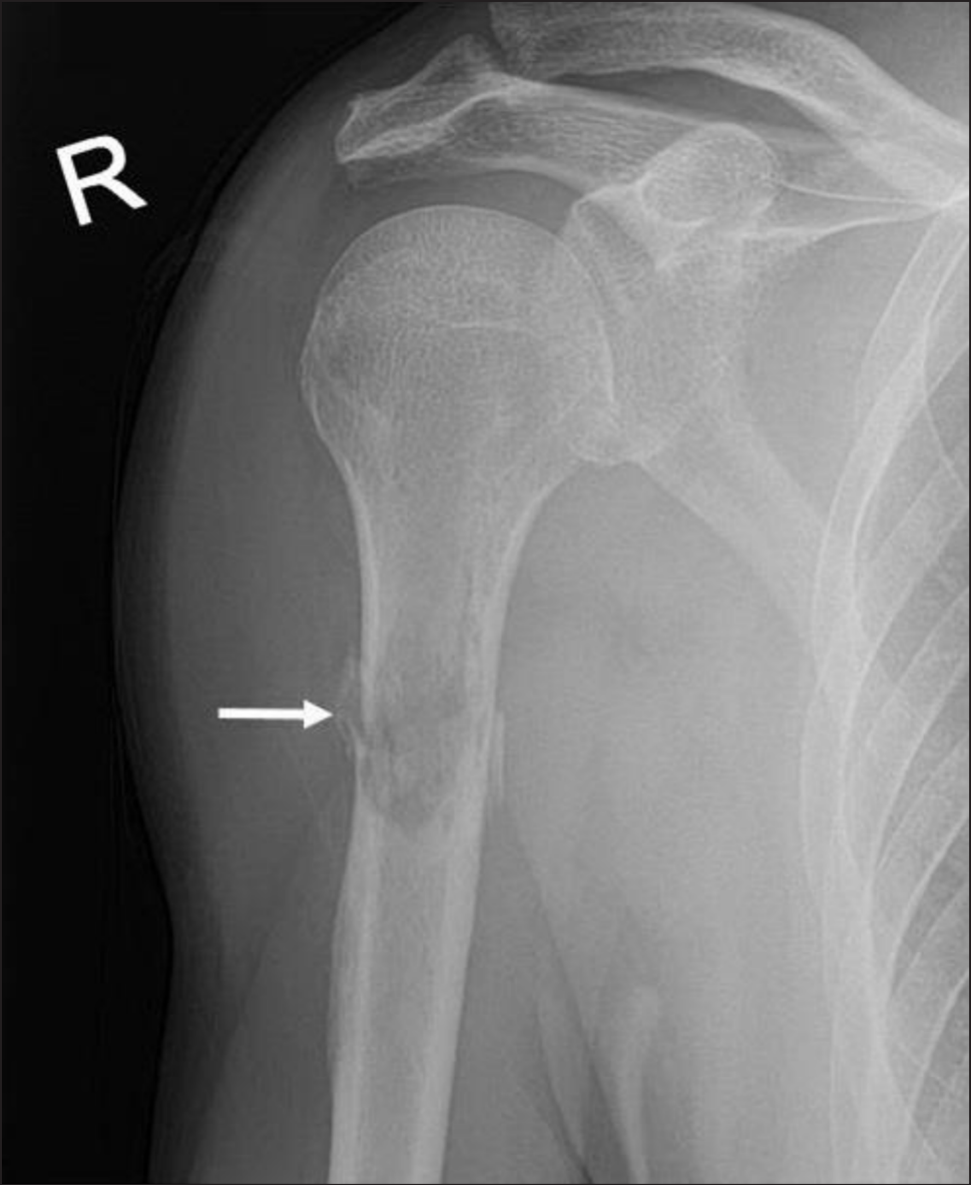
X-ray of the humerus: Fracture of the proximal humeral diaphysis (arrow) with a hazy appearance and a wide transition zone.

**Figure 2:**
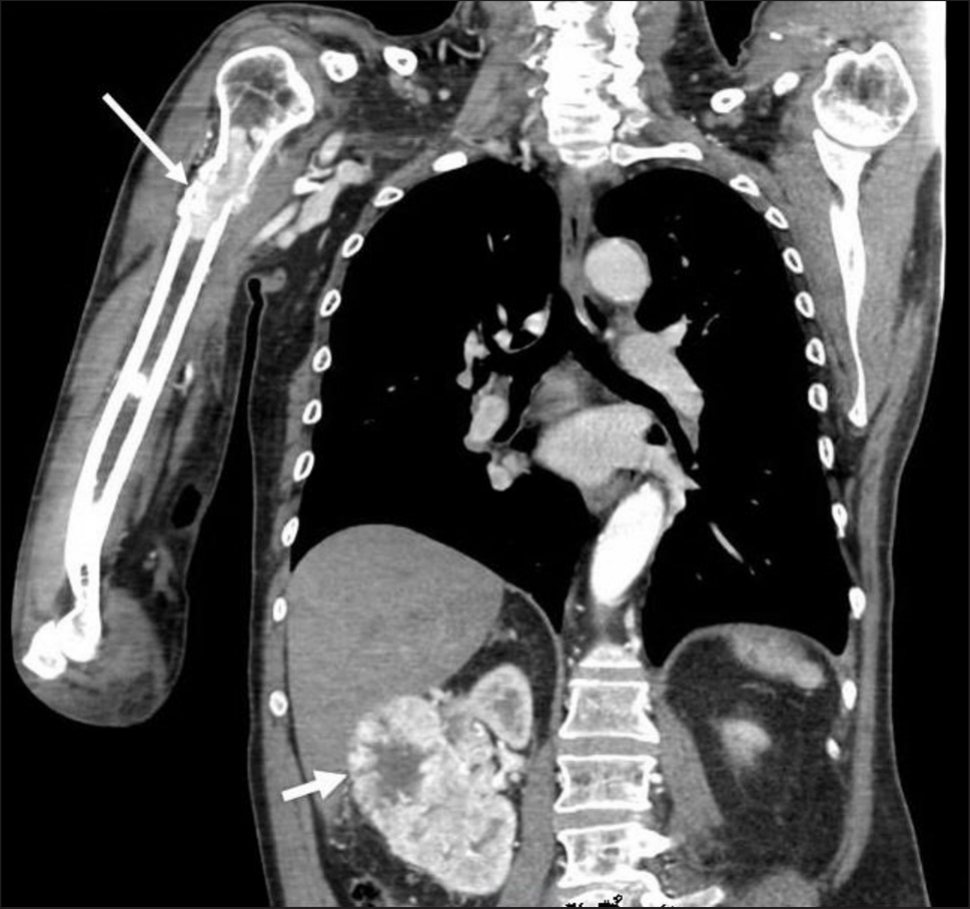
CT of thorax and abdomen: Enhancing lesion at the site of the fracture (long arrow). Exophytic mass at the mid and lower pole of the right kidney (short arrow).

**Figure 3:**
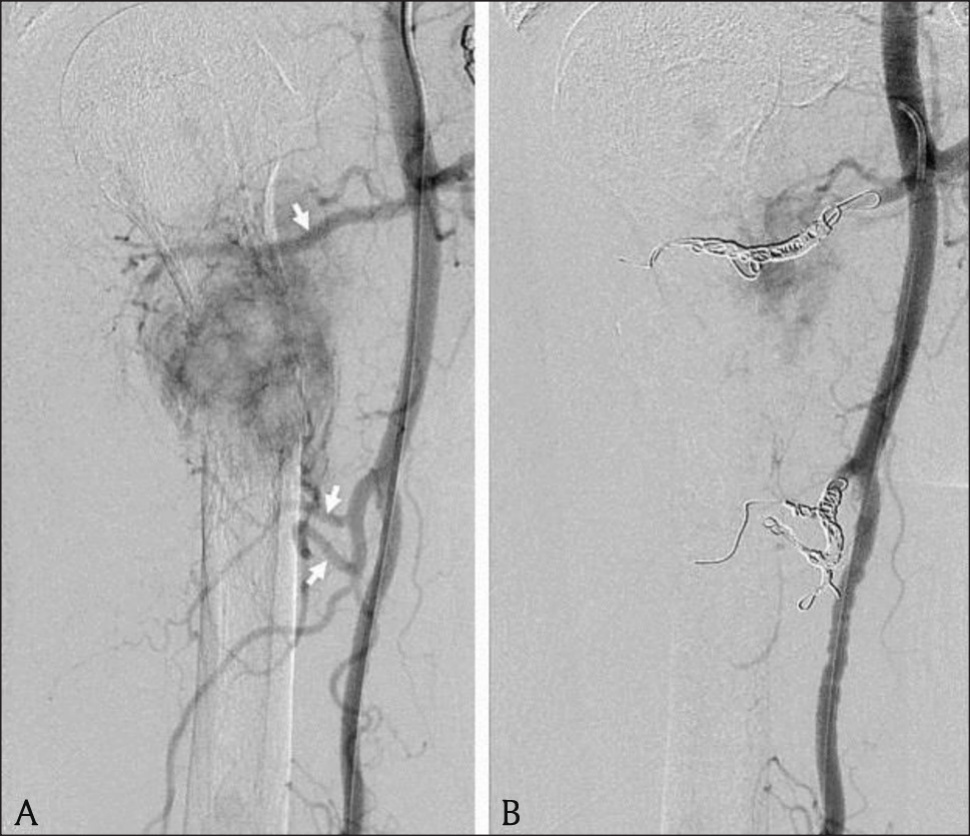
Angiography. **3A:** Three feeding branches (arrows) arising from the axillary artery to the bone metastasis. **3B:** Endovascular embolization of the feeding branches with coils.

A 68-year-old man presented with atraumatic tenderness of the right upper arm. An X-ray demonstrated a fracture of the proximal humeral diaphysis with a hazy appearance and a wide transition zone, raising suspicion of a pathologic fracture (Figure 1). On computed tomography (CT) of thorax and abdomen, there was an enhancing lesion at the site of the fracture. An exophytic mass at the mid and lower pole of the right kidney was also demonstrated (Figure 2). Findings were in keeping with a renal cell carcinoma (RCC) with hypervascular bone metastasis resulting in a pathologic fracture, which demanded intramedullary nailing. Because of significant risk of operative bleeding from the bone metastasis, an angiography with endovascular embolisation was performed. On angiography, at least three feeding branches arising from the axillary artery to the bone metastasis at the fracture site were identified (Figure 3A). Due to extensive venous shunting, particles could not be used for embolization. The feeding vessels were embolised using coils (Figure 3B). Surgery on the fracture was subsequently performed with no notable bleeding.

## Discussion

Metastasis is the most common malignant lesion of the bone and is associated with significant morbidity. Symptoms of bone metastasis, such as pain or impending fracture, are often treated with medical therapy or radiotherapy, with variable results. In the past few years, interventional radiology (IR) has taken up an increasing role in the management of bone metastasis. IR can be used as a curative treatment in selected patients with oligometastatic disease or as palliative strategy, most often as pain relief. IR offers several advantages, such as a synergic effect with all other treatments, no need for interruption of systemic tumour therapy, reduced morbidity and in-hospital stay, and fast recovery. The decision to perform an IR procedure in a patient with bone metastasis has to be taken in consensus by a multidisciplinary tumour board. Most common IR procedures of bone metastasis are osteoplasty, osteosynthesis, thermal ablation, and embolisation. Trans-arterial embolisation (TAE) is the interventional technique in which hypervascular bone metastases are selectively devascularised with preservation of all non-target vessels. Possible indications for reducing tumour vascularity with TAE are to minimize blood loss during surgery, to relieve pain, to reduce the risk of spontaneous bleeding, and to limit the heat sink effect from surrounding vessels during thermal ablation. Renal function, coagulation state, and inflammatory parameters should be addressed before procedure. The interventional technique depends on indication, vessels, and regional anatomy. Embolic agents can be temporary or permanent and liquid or solid. Permanent agents are most commonly used in a preoperative or a palliative setting. Liquid agents, often combined with embolisation coils or plugs, are applied in the presence of arterio-venous shunting. Particles are mainly used for bone devascularisation [1].

In conclusion, IR can offer unique advantages in the treatment strategy of the often complex clinical scenario of bone metastasis. Several procedures are available and are chosen based on the assessment by a multidisciplinary tumour board.
